# Medicinal compounds and biotechnology of Amaryllidaceae alkaloids in *Lycoris radiata*

**DOI:** 10.3389/fpls.2025.1639654

**Published:** 2025-12-15

**Authors:** Jia-Xu Chen, Jiafeng Zheng, You-Ming Cai, Jun-Xu Xu, Yi Sun, Zhen Yang, Feng Yang, Mo-Xian Chen, Yu Chen, Qing-Zhu Li

**Affiliations:** 1 State Key Laboratory for Development and Utilization of Forest Food Resources, State Key Laboratory of Tree Genetics and Breeding, The Southern Modern Forestry Collaborative Innovation Center, College of Life Sciences, Nanjing Forestry University, Nanjing, China; 2 Forestry and Pomology Research Institute, Shanghai Key Laboratory of Protected Horticultural Technology, Shanghai Academy of Agricultural Sciences, Shanghai, China; 3 Shenzhen Research Institute, The Chinese University of Hong Kong, Shenzhen, China

**Keywords:** *Lycoris radiata*, Amaryllidaceae alkaloids, biosynthesis, medicine value, alkaloid

## Abstract

*Lycoris radiata*, known for its striking floral patterns and vivid colors, holds significant ornamental value and is widely admired by the public. As research on *Lycoris* species progresses, scientists have uncovered their significant medicinal potential. These plants are particularly valued for their alkaloid compounds, which exhibit important pharmacological properties, especially strong antibacterial effects. This study systematically investigates the medicinal properties of *Lycoris* alkaloids. Through a comprehensive review, we analyze the various types of alkaloids present in *Lycoris* species. It sheds light on their synthetic mechanisms and elucidates their multifaceted functions, providing a detailed understanding of their pharmacological potential. Moreover, this paper highlights recent breakthroughs in alkaloid research, presenting the latest advancements in this field. By systematically documenting and elucidating these aspects, this study aims to provide a comprehensive understanding of the medicinal value of *Lycoris* and the intricate roles played by its alkaloid constituents.

## 1 Introduction

### 1.1 Introduction to *Lycoris*

*Lycoris*, a perennial monocotyledonous herb of the genus Lycoris (Amaryllidaceae), is native to subtropical East Asia, including China, Korea, Japan, and Taiwan (Tae and Go, 1995).This genus contains approximately 20 species, with 15 present in China (Tae and Ko, 1993). These species are predominantly found in the southern region of the Yangtze River, particularly Jiangsu, Anhui, and Zhejiang Provinces. *Lycoris* plants typically thrive in damp and cool environments, such as brook rocks. Their bulbs are covered with dark brown membranous scales, and their leaves are flat and elongated, resembling narrow strips. This morphological similarity to garlic explains the origin of the genus name “*Lycoris*”. First described by Herbert in 1821 (Ping-sheng et al., 1994), the genus has gained research significance, with *Lycoris aurea* becoming a model organism because of its distinctive traits and high interspecific cross-compatibility (Zhang et al., 2020). The frequent natural hybridization within the genus offers valuable opportunities to study the genetic and physiological diversity of Lycoris (Lv et al., 2023). *Lycoris radiata* (L’Hér.) Herb., commonly referred to as red spider lily or stone garlic, has been extensively utilized in traditional Chinese medicine (TCM) as an ethnopharmacologically significant plant (Liao et al., 2012). The bulbs of *L. radiata* contain a variety of bioactive Amaryllidaceae alkaloids, which have been employed in folk medicine for the management of neurological disorders, including Alzheimer’s disease (AD), other neurodegenerative conditions, and poliomyelitis (Chen et al., 2016; Hu et al., 2018; Shen et al., 2019). As documented in the *Compendium of Materia Medica* (*Ben Cao Gang Mu*), *L. radiata* exhibits therapeutic properties such as detoxification, analgesia, anti-inflammatory effects, and diuresis, and has been traditionally applied in the treatment of abscesses, suppurative wounds, and ulcers (Jin and Yao, 2019). Modern pharmacological studies have validated these traditional uses, demonstrating that its active components (e.g., lycorine and galanthamine) possess neuroprotective, anti-inflammatory, and antimicrobial activities (Shen et al., 2019).

In recent years, *Lycoris* has attracted considerable research interest owing to its medicinal potential, which is primarily attributed to the presence of Amaryllidaceae alkaloids in its bulbs (Ding et al., 2017). These alkaloids demonstrate diverse pharmacological activities, including antimalarial (Hao et al., 2013; Toriizuka et al., 2008), antiviral (Szlávik et al., 2004; Zou et al., 2009), and immunostimulatory effects (Liao et al., 2012). Notably, isoquinoline alkaloids from the Amaryllidaceae family have shown significant potential in drug discovery and development. These compounds have been traditionally used for their antiseptic and wound-healing properties, as well as for treating alcoholism (Nair and van Staden, 2019). Notably, galantamine, a representative alkaloid derived from *Lycoris*, has been clinically approved for treating mild-to-moderate AD (Howes and Houghton, 2003). Another key compound, lycorine, exhibits broad-spectrum bioactivities including antibacterial, antimalarial, and cardioprotective effects. These bioactive constituents highlight *Lycoris* as a promising source for natural drug development (Zhao et al., 2023).

With the intensification of ageing of the global population, the pathology of neurodegenerative diseases has increased, leading to a growing demand for galantamine (Wang et al., 2024). However, the complex structure of galantamine poses significant challenges in chemical synthesis, making large-scale commercial production through total chemical synthesis difficult (Yeo et al., 2021). Consequently, the extraction of natural galantamine from *Lycoris* bulbs has become increasingly important. Chinese researchers reported in 2014 that global galantamine production (300–400kg/year) met less than 0.2% of the annual worldwide demand (175,000–263,000 kg/year). Currently, *Lycoris* serves as the primary source for galantamine production in China, resulting in rapidly growing market demand(X. Chang, 2015).

China possesses abundant species diversity in the *Lycoris* genus, yet most populations remain in wild conditions (Zhang et al., 2021). The rising market demand for cut flowers and for medicinal purposes has caused excessive bulb harvesting, posing severe threats to wild populations because of habitat destruction. Additionally, the natural reproduction of *Lycori*s faces biological constraints. Its regeneration efficiency in the wild is low—typically, only one or two axillary buds sprout and form bulbs annually (Ren et al., 2022). The long growth cycle further hinders commercial-scale cultivation. Although tissue culture techniques provide partial solutions, traditional micropropagation systems still face multiple challenges. These include callus browning, vitrification, microbial contamination, low induction/proliferation efficiency, and poor acclimatization resulting in low transplant survival rates (Zhang et al., 2024).

While more than 230 *Lycoris* cultivars are currently used as ornamental garden plants, their alkaloid concentrations remain extremely low. For instance, less than 0.1% galantamine is present in *Lycoris radiata*. Consequently, the demand for *Lycoris* bulbs remains high. Beyond meeting this demand, enhancing the alkaloid content represents another key research focus. Studies indicate that the alkaloid levels in the same species vary across habitats, and researchers have explored the introduction of endophytic fungi to *Lycoris* bulbs to increase alkaloid production (Wang et al., 2024).

### 1.2 Alkaloid species in Lycoraceae

**Figure 1:**
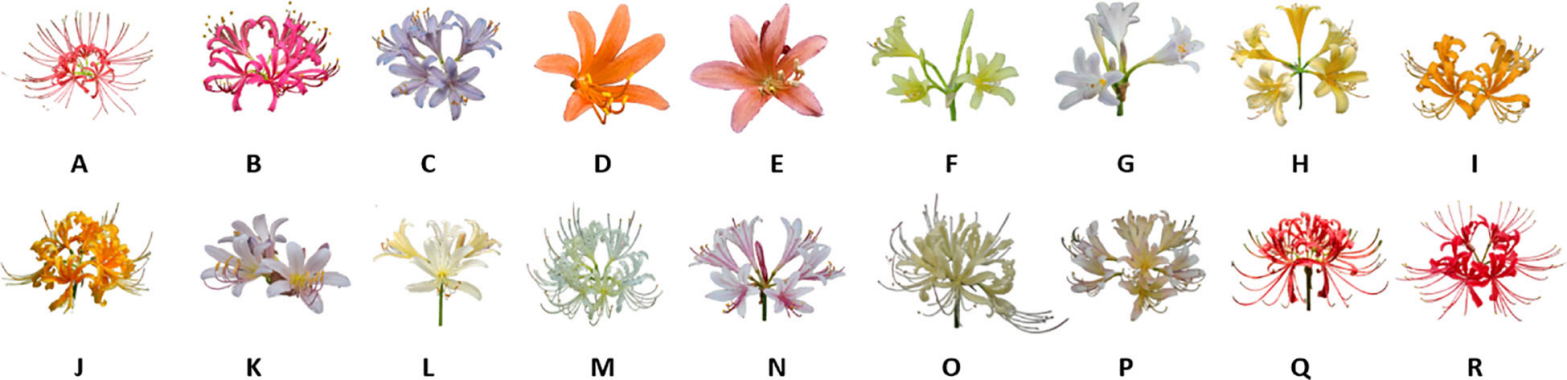
Diversity and characteristics of 18 species of Lycoris. **(A)***Lycoris radiata var. pumila,***(B)***Lycoris rosea,***(C)***Lycoris sprengeri,***(D)***Lycoris sanguinea var. sanguinea,***(E)***Lycoris sanguinea koreana,***(F)***Lycoris longituba var. flava,***(G)***Lycoris longituba,***(H)***Lycoris anhuiensis,***(I)***Lycoris chinensis,***(J)***Lycoris aure,***(K)***Lycoris chinensis x Lycoris sprengeri,***(L)***Lycoris caldwellii,***(M)***Lycoris albifora,***(N)***Lycoris incarnata,***(0)***Lycoris straminea,***(P)***Lycoris flavescens,***(Q)** Lvcoris radiata, **(R)***Lycoris chunxiaoensis*.

The *Lycoris* genus is widely recognized for its diverse alkaloid profiles, with hundreds of compounds having been isolated and characterized (Cahlíková et al., 2020). As documented in the *Flora of China* (Fennell and van Staden, 2001), the genus has 15 species in China, including *L. albiflora*, *L. anhuiensis*, *L. aurea*, *L. caldwellii*, *L. chinensis*, *L. guangxiensis*, *L. houdyshelii*, *L. incarnata*, *L. longituba*, *L. radiata*, *L. rosea*, *L. shaanxiensis*, *L.* sp*rengeri*, *L. squamigera*, and *L. straminea*. These species possess both ornamental and medicinal value. This study focuses on the morphological characterization of 18 representative *Lycoris* species. (**Figure 1**).

**Figure 2:**
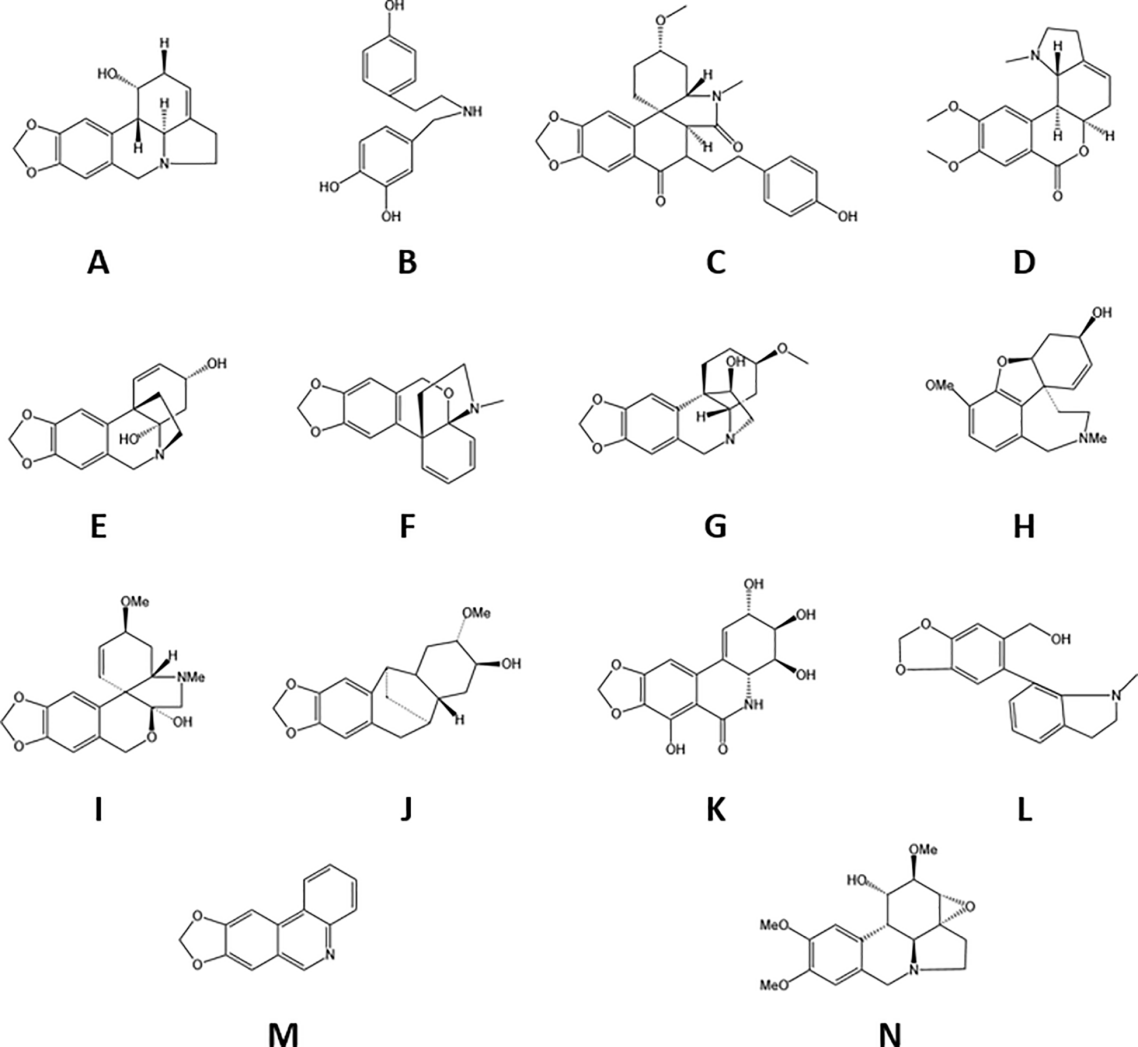
The species of alkaloids in Lycoraceae. **(A)***Lycorine*, **(B)***Norbelladine*, **(C)***plicamine*, **(D)***homolycorine*, **(E)***crinine*, **(F)***graciline*, **(G)***haemanthamine*, **(H)** galanthamine, **(I)***galanthindole*, **(J)***phenanthridone*, **(K)***tazettine*, **(L)** montanine, **(M)***miscellaneous*, **(N)***phenanthridine*.

*Lycoris* alkaloids, which are tyrosine-derived compounds predominantly biosynthesized by the Amaryllidaceae family, represent a structurally diverse group of plant metabolites. Current research has identified 636 distinct alkaloids from *Lycoris* species, with complete structural elucidation achieved for more than 100 compounds (Jin and Yao, 2019). These nitrogenous secondary metabolites are categorized into 18 major structural classes: norbelladine-, lycorine-, homolycorine-, galasine-, galanthindole-, crinine-, haemanthamine-, cripowellin-, narciclasine-, pretazettine-, plicamine-, secoplicamine-, graciline-, montanine-, galanthamine-, ismine-, and Sceletium-type alkaloids, along with miscellaneous derivatives (Georgiev et al., 2024). Of particular importance is galanthamine, which has been rigorously investigated as a potential therapeutic agent for AD (Marco-Contelles et al., 2006). Representative alkaloid structures are presented in **Figure 2**. This remarkable structural diversity positions Lycoris alkaloids as valuable candidates for pharmaceutical development.

### 1.3 Systematic review of alkaloids in the *Lycoris* genus

#### 1.3.1 Chemical diversity of lycorine alkaloids in *Lycoris* species

**Figure 3:**
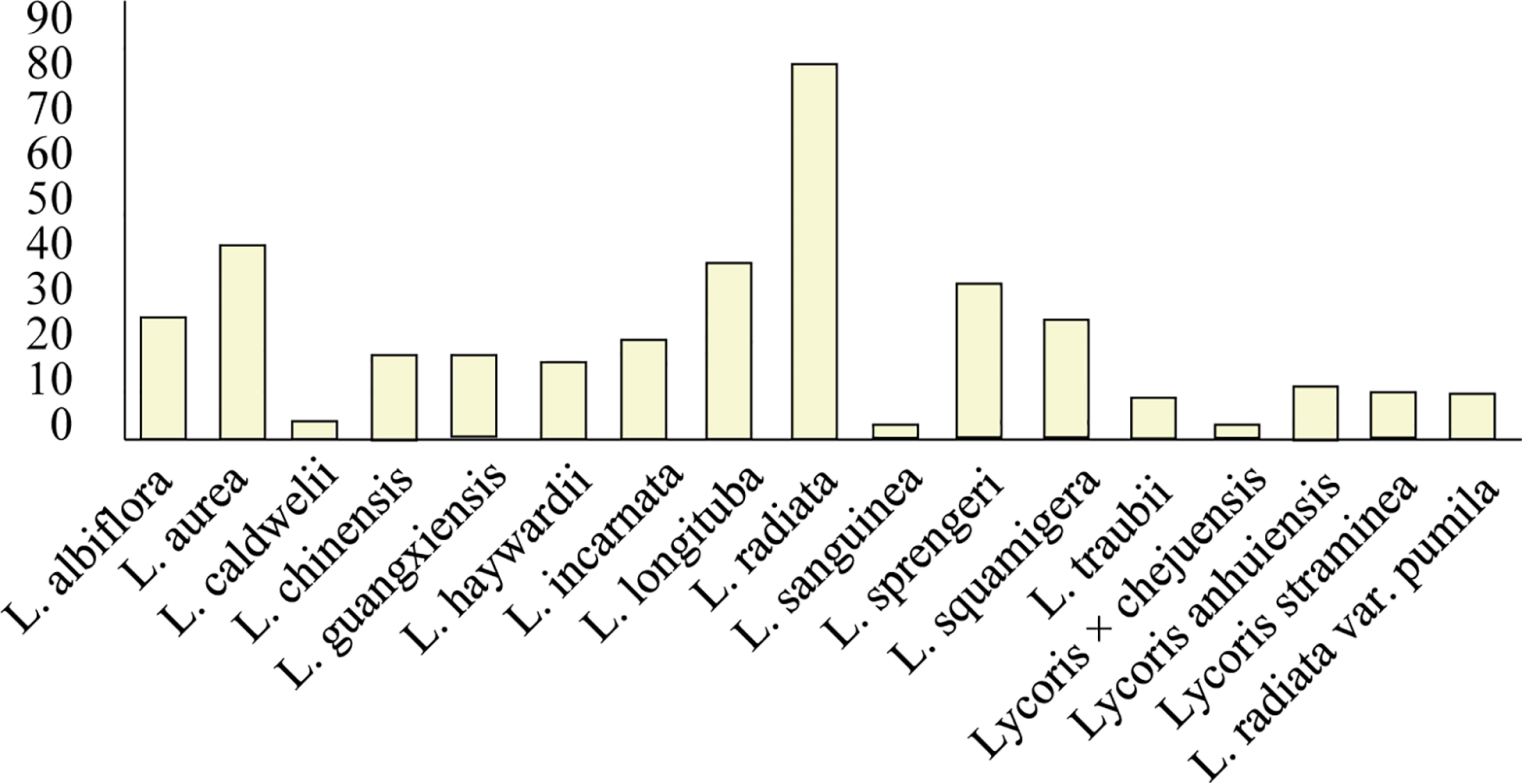
Quantification of identified alkaloids across Lycoris species. *L. radiata* demonstrates the highest alkaloid diversity (83), with *L. aurea* (3) and *L. longituba* (39) showing secondary abundance. Values represent total characterized alkaloids per species. Species names are on the x-axis, and alkaloid content is on the y-axis.

Owing to its economic significance, ornamental value, and potential therapeutic applications for neurological disorders, the Lycoris genus has become increasingly important in the Amaryllidaceae family (Ren et al., 2021, Ren et al., 2022; Zhou et al., 2020). The genus derives its common name from the bulb’s morphological similarity to garlic. However, its taxonomic classification remains challenging because of widespread interspecific hybridization, which has produced numerous hybrids with highly similar morphological characteristics among varieties (Quan et al., 2024). According to Plants of the World Online (POWO);, 29 *Lycoris* species are currently documented, with 25 being taxonomically accepted. Our alkaloid research analysis revealed that the alkaloid contents of 18 species, which produce approximately 120 characterized alkaloids, have been studied (**Figure 3**, **Supplementary Table 1**). Notably, *L. radiata* var. *pumila* is recognized as a distinct variety, whereas *L. anhuiensis* and *L. straminea* have been reported in only a single Chinese study. Among the investigated species, *L. radiata* (red spider lily) has the most diverse alkaloid profile and is the most extensively studied taxon. Several other species—including *L. aurea*, *L. longituba*, *L.* sp*rengeri*, *L. squamigera*, and *L. incarnata*—also demonstrate considerable alkaloid diversity and abundance (Cahlíková et al., 2020).

Among the 130 alkaloids identified in *Lycoris* species, only 7-deoxy-trans-dihydronarciclasine(138) (exclusively found in *L. chejuensis*) represents a novel discovery, as all others have been documented in previous reviews (Cahlíková et al., 2020). These alkaloids can be categorized into 16 core structural types (Lin et al., 2025), among which the lycorine-type is the most abundant, being found in 26% of all characterized compounds. Galanthamine-type alkaloids have been the most extensively studied because of their clinical use in AD treatment. Notably, hostasinine-type alkaloids are currently represented solely by hostasinine A, which has been identified only in *L. albiflora* (Jitsuno et al., 2011). Similarly, the galanthindole-type is restricted to lycosprinine B (*L.* sp*rengeri*) (Wu et al., 2014), and the ismine-type is restricted to ismine (*L. squamigera*) (Kitajima et al., 2009).

#### 1.3.2 Organ-specific distributions of alkaloids

Plant growth is a dynamic process characterized by stage-dependent expression patterns of secondary metabolites. In medicinal plants, bioactive compounds have distinct organ-specific distributions, as demonstrated by multiple studies. *Lycoris* species, known for their pharmacologically active alkaloids including galantamine, lycorine, and lycoramine, display significant spatiotemporal variations in secondary metabolite accumulation. These variations have important implications for both plant defense systems and pharmaceutical development. Research has indicated that in Lycoris chinensis (Mu et al., 2010), mature seeds exhibit the highest galantamine content (671.33 µg/g DW), which is 5.2 times greater than that in leaves, while lycorine primarily accumulates in root hairs (505.85 µg/g DW) and lycoramine in seed coats (383.62 µg/g DW). This compartmentalization pattern suggests functional adaptation: elevated alkaloid levels in seeds may provide chemical defense for embryos, whereas root accumulations likely protect against soil pathogens. Notably, the alkaloid content followed a U-shaped developmental pattern, with the highest levels in mature seeds, followed by perennial plants, 1-year-old seedlings, and 3-month-old seedlings, and the lowest in 10-day-old seedlings. Metabolomic and transcriptomic analyses of *L. radiata* (Park et al., 2019), further confirmed organ-specific variations. HPLC quantification revealed a ubiquitous galantamine distribution, with concentrations of 0.53 ± 0.07 mg/g DW in roots, 0.27 ± 0.04 mg/g DW in bulbs, and 0.75 ± 0.09 mg/g DW in leaves. Bulb galantamine levels were 1.42- and 2.78-fold higher than those in roots and leaves, respectively, corresponding to upregulated expressions of biosynthetic genes (e.g., *LrNNR, LrN4OMT*, and *LrCYP96T*). In contrast, *L. longituba* (Li et al., 2020) exhibited a maximum galantamine accumulation in roots (976.14 mg/g DW versus 263.51 mg/g DW in bulbs and 61.63 mg/g DW in leaves), accompanied by tissue-specific expressions of pathway genes (root-predominant OMT versus bulb-specific CYP96T1). This interspecies variation underscores the metabolic diversity within the genus, although current studies have yet to fully characterize the organ-specific alkaloid distribution patterns across *Lycoris* taxa.

#### 1.3.3 Extraction and detection techniques for lycorine alkaloids

Various extraction methods have been employed to isolate *Lycoris* alkaloids, with solvent extraction using methanol or ethanol remaining the most conventional approach (Song et al., 2014; Naidoo et al., 2021). Currently, the majority of alkaloid studies utilize alcoholic extracts (Zhou et al., 2024), which are frequently combined with auxiliary techniques to improve extraction efficiency and detection sensitivity. For example, methanol-based ultrasound-assisted extraction (using a 1:10 sample-to-solvent ratio with 45 minutes of ultrasonication) coupled with UHPLC – QTOF – MS/MS analysis successfully identified 37 alkaloids, including 16 novel compounds, with detection limits at the nanogram level. Significant interspecific variation in alkaloid profiles were observed among *Lycoris* species, with *L.* sp*rengeri* showing the highest alkaloid diversity and concentration, indicating considerable medicinal potential (Sun et al., 2017). In another study *L. radiata* bulbs were extracted using 70% ethanol, yielding 212 g of extract from 5 kg of raw material (4.24% extraction yield). Subsequent chloroform fractionation resulted in 80.2% enrichment, followed by multistep chromatography (silica gel, RP-18, and Sephadex LH-20) to isolate nine alkaloids (35–98 mg each). Notably, yemenine A (139) was obtained with a yield of 0.00196% (98 mg/5 kg) (Hu et al., 2018). Recent methodological advances integrate ultrasound-assisted extraction with capillary electrophoresis-electrochemiluminescence (CE-ECL), enabling baseline separation of four alkaloids within 16 minutes and achieving detection limits of 1.8–14 ng/mL. Compared with conventional methods, this technique has three distinct advantages: (1) a 46-minute reduction in analysis time, (2) nanogram-level sensitivity, and (3) microliter-scale solvent consumption, thereby establishing an efficient approach for trace alkaloid analyses in complex matrices (Sun et al., 2017). A novel solid-phase extraction (SPE) technique utilizing electrostatic repulsion principles was developed for alkaloid extraction from *Lycoris* and four related plant species, with subsequent LC – MS analysis. Under optimized acidic conditions (0.1% formic acid, pH 3.0), this method achieved the selective elution of positively charged alkaloids (10% methanol) while retaining nonalkaloid components through hydrophobic interactions (90% methanol). LC – MS analysis successfully identified 42 intact alkaloids, overcoming the structural degradation issues associated with pH fluctuations associated with conventional approaches (Du et al., 2019). These methodological advances establish a robust foundation for the quality control and pharmaceutical development of *L. radiata* and related species.

#### 1.3.4 Factors influencing alkaloid contents in *Lycoris* species

The alkaloid contents in *Lycoris* plants are influenced by multiple interacting factors that collectively determine their medicinal and economic value. Alkaloid accumulations in this genus arise from complex interactions among genetic factors, environmental conditions, and endophytic microbial communities. Comparative analyses demonstrated significant interspecific variation in alkaloid content among *Lycoris* species, with *L. radiata* (0.48 mg/g DW) exhibiting substantially higher levels than *L. chinensis* (0.18 mg/g DW) and other examined species including *L. sanguinea*, *L. squamigera*, *L. uydoensis*, and *L. chejuensis* (Yeo et al., 2021). In addition to quantitative variations, the alkaloid profiles differ significantly among *Lycoris* species. UHPLC – QTOF – MS/MS analyses of *L. radiata*, *L. aurea*, *L. rosea*, *L. straminea*, *L.* sp*rengeri*, and *L. longituba* revealed 37 alkaloids. The structural diversity and relative abundance for L. sprengeri were greatest, whereas those of *L. straminea* were the lowest. Notably, *L. radiata* and *L. aurea* accumulate relatively high concentrations of polar alkaloids ( Li et al., 2019), underscoring the pharmaceutical potential of this genus.

Geographical distribution and environmental conditions significantly influence alkaloid accumulation patterns. Intraspecific variations are evident across regions, as shown by *L. aurea* populations from Jiangxi Quannan containing 6–7 times higher lycorine and galantamine levels than those from Hunan Zhongfang (**Table 1**). These alkaloid levels are positively correlated with specific soil parameters (Zuo et al., 2025). Despite their pharmacological importance, most alkaloids in wild *Lycoris* plants are present in trace quantities (<0.1% dry weight for galantamine), which restricts their commercial utilization (Liu et al., 2020). Emerging evidence indicates that *Lycoris*-endophyte interactions modulate alkaloid biosynthesis through complex regulatory networks (Zhou et al., 2020). Tissue-specific endophytic communities have been characterized across different plant organs (Liu et al., 2020). Endophytic diversity and regulatory functions vary significantly among *Lycoris* species.*L. aurea* maintained higher fungal diversity than *L. radiata* did, with enriched taxa (e.g., *Phyllosticta*, *Colletotrichum*, and *Acrocalymna*) showing positive correlations with galantamine accumulation. Notably, endophytes isolated from *L. aurea* (e.g., *Phyllosticta* sp. LaL1, *Colletotrichum* sp. LaR6, *Clonostachys* sp. LaR14, and *Acrocalymna* sp. LaR15) selectively increased galantamine production (but not lycorine production) in *L. radiata*. These findings demonstrate the cross-species applicability of endophytic inoculants and establish a theoretical foundation for *Lycoris* microbiome research (Wang et al., 2024).

**Table 1:** Mechanism of action of drugs related to Alzheimer’s disease.

Drug name	Mechanism of action	Interaction type	references
Galanthamine	Competitive Inhibition	Reversible AChE Inhibition	(Metz and Pavlov, 2021) (Park et al., 2019) (Hafiz et al., 2023)
Donepezil	Competitive Inhibition	Reversible AChE Inhibition	(Marucci et al., 2021) (Guo et al., 2020) (Rong et al., 2021)
Rivastigmine	Non-competitive Inhibition	Non-reversible AChE Inhibition	(Kandiah et al., 2017) (Reilly et al., 2023)

This table summarizes the names, mechanisms of action, and interaction types of several alkaloids. Galantamine serves as a competitive inhibitor, capable of reversibly inhibiting acetyl-cholinesterase (AChE), and its mechanism has been validated in multiple studies. Donepezil also acts as a competitive inhibitor with reversible effects on AChE. Rivastigmine, on the other hand, is a non-competitive inhibitor that irreversibly inhibits the activity of AChE. Detailed references for each drug are listed in the corresponding reference section.

## 2 Pharmacological activities of alkaloids from Lycoris species

### 2.1 Cholinesterase inhibition

**Figure 4:**
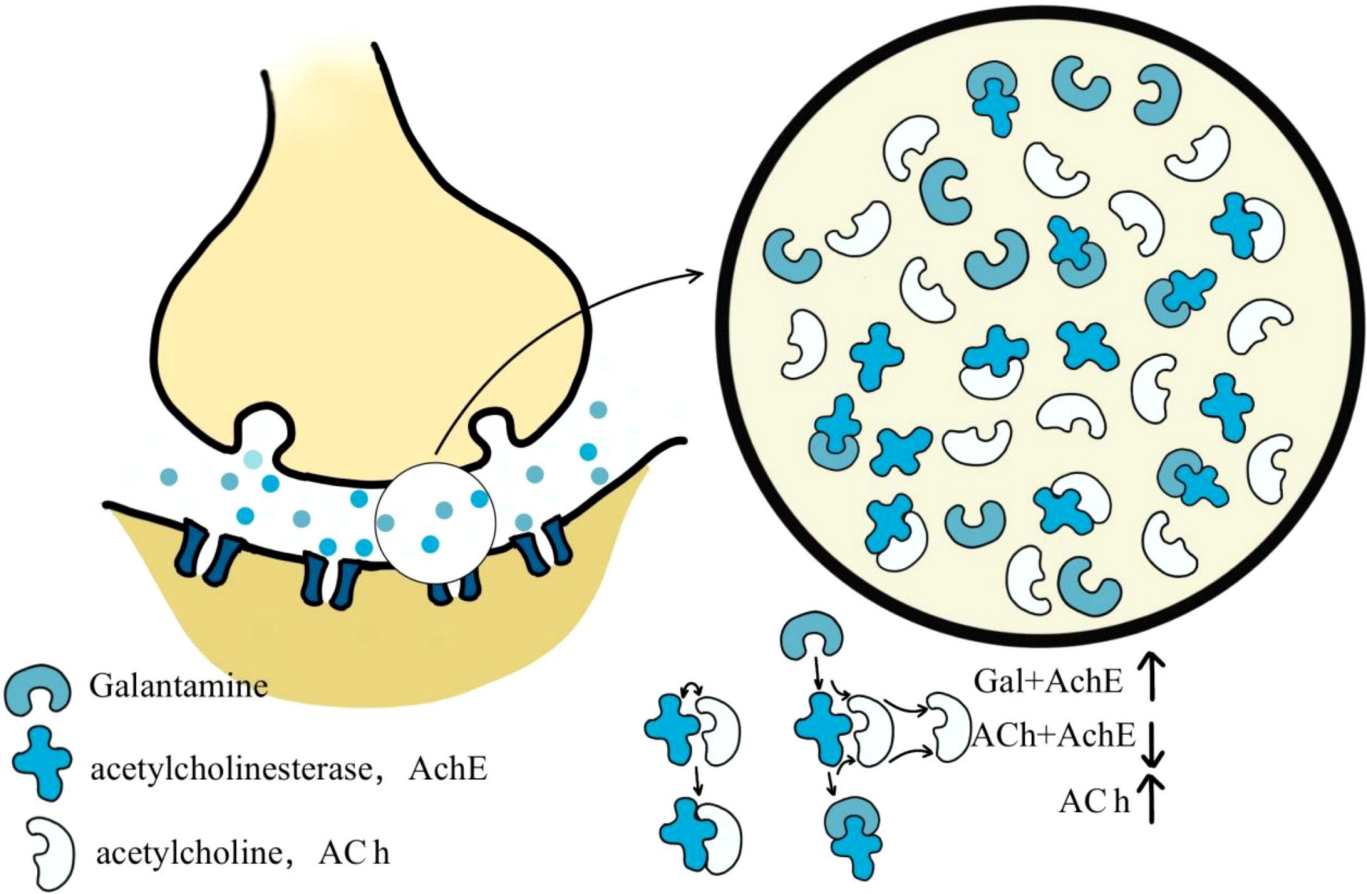
Mechanism of action of galanthamine Galantamine is a specific, competitive, and reversible acetylcholinesterase (AChE) inhibitor. It competes with acetylcholine (ACh) at the synaptic cleft, preventing the degradation of ACh by inhibiting its binding to acetylcholinesterase.

The *Lycoris* genus is widely recognized for producing galanthamine, a natural alkaloid clinically used to treat AD. With the molecular formula C_17_H_21_NO_3_ (MW: 323.8145), this secondary metabolite isolated from *Lycoris* plants functions as an approved acetylcholinesterase (AChE) Inhibitor in medical practice (2012) (**Figure 4**). Galantamine acts as a selective, reversible, and competitive AChE inhibitor (Desgagné-Penix, 2021) (**Table 1**). Galantamine acts as a selective, reversible, and competitive AChE inhibitor.

Lycoramine (dihydrogalantamine, C_17_H_23_NO_3_), initially isolated from Amaryllidaceae bulbs in the 1950s, is another effective AChE inhibitor. This compound has been clinically applied in the treatment of myasthenia gravis and postpolio syndrome (Irwin and Smith, 1960). Recent investigations of Chinese *Lycoris* species have indicated that the content of lycoramine is approximately 7-fold greater than that of galanthamine (Tang et al., 1964). Preliminary clinical trials in China have shown that compared with galanthamine lycoramine hydrobromide effectively alleviates muscle weakness, albeit with marginally lower efficacy. Importantly, compared with galanthamine (Uyeo and Koizumi, 1953), lycoramine enhances striated muscle contraction through cholinesterase inhibition, resulting in a therapeutic index similar to that of galanthamine, suggesting the potential for expanded clinical use.

### 2.2 Antiviral activity

Lycorine demonstrates broad-spectrum antiviral activity against diverse viruses. A 2011 Jiangning study reported the effective inhibition of enterovirus 71 (EV71), a single-stranded positive-sense RNA virus (Picornaviridae family), by lycorine treatment. This study revealed that lycorine significantly decreased mortality in EV71-infected mice by blocking viral protein synthesis (Liu et al., 2011), indicating its potential utility for managing EV71 outbreaks in Asia. In 2020, Chen et al. reported the inhibitory effect of lycorine on Zika virus (ZIKV). Originally discovered in 1947 during yellow fever surveillance in Uganda’s Zika Forest (Chen et al., 2020), ZIKV infection is clinically associated with Guillain–Barré syndrome, meningitis, and other neurological complications. Their research demonstrated that lycorine effectively suppressed ZIKV replication *in vitro* through specific inhibition of the viral RNA-dependent RNA polymerase (RdRp), highlighting its therapeutic potential.

Lycorine exhibits antiviral activity against the highly pathogenic avian influenza H5N1 virus (Yang et al., 2019). In screening anti-coronaviral alkaloids, pancracine (96), 6β-acetyl-8-hydroxy-9-methoxycrinamine (26), haemanthamine (29), and haemanthidine(30) showed efficacy comparable to that of lycorine in suppressing human coronavirus (HCoV)-OC43 replication (Merindol et al., 2024). Additionally, lycoricidine derivatives from *Lycoris radiata* extracts demonstrated notable activity against tobacco mosaic virus (TMV), with *N*-methyl-2,3,4-trimethoxylycoricidine (131) and *N*-methyl-2-methoxy-3,4-acetonidelycoricidine (132) having the strongest inhibitory effects (Yang et al., 2018).

### 2.3 Antitumor activity

Lycorine is an isoquinoline alkaloid that is abundant in Amaryllidaceae plant bulbs. It exerts antitumor effects through three main mechanisms: inhibiting tumor cell metastasis, inducing tumor cell apoptosis, and arresting the cell cycle. Studies have shown that lycorine downregulates the key metastatic regulators, MMP-9 and MMP-2 in a dose-dependent manner, thereby suppressing cell migration (Liu et al., 2018). In 2019, Ning et al. demonstrated that lycorine induces reactive oxygen species (ROS)-mediated activation of the p38 MAPK (mitogen-activated protein kinase) and p53-dependent apoptotic pathways in human osteosarcoma cells, leading to G1 phase arrest without causing organ toxicity (Ning et al., 2020). Lycoreine (55) (C_18_H_23_NO_4_; molecular weight: 317.38), a pyrrole-type alkaloid extracted from *Lycoris* species (e.g., *L. radiata* and *L. squamigera*), shares a common biosynthetic precursor 4’-O-methylnorbelladine with other *Lycoris* alkaloids (Ding et al., 2017). This compound demonstrates notable cytotoxicity against leukemia and hepatocellular carcinoma cells, showing approximately twofold higher potency than its maximum nontoxic concentration (MNTC) and superior efficacy to homolycorine(49). Its antitumor mechanisms involve inhibition of topoisomerase I, DNA breakage and recombination, and induction of apoptosis (Nair and Van Staden, 2021). Although it enhances chemosensitivity and reduces drug resistance, compared with other Lycoris alkaloids, lycoreine(55) is less effective against hepatic, uterine, and breast cancers (Jia et al., 2024; Yang et al., 2024).

The total alkaloid extracts of *L. aurea*, *L. radiata*, and *L. guangxiensis* significantly inhibited the growth of HepG2 cells, with *L. radiata* extract showing 84.91% inhibition (Tian et al., 2015a). Notably, (+)-N-methoxylcarbonyl-nandigerine(126) and (+)-N-methoxycarbonyl-lindcarpine(127) isolated from *L. caldwellii* exhibited potent cytotoxicity against astrocytoma CCF-STTG1 and glioma (e.g., CHG-5, SHG-44, and U251) cell lines, with IC50 values ranging from 9.2 to 12.2 μM (Cao et al., 2013), suggesting therapeutic potential for nervous system tumors. Furthermore, 2-demethyl-isocorydine, (+)-8-hydroxy-homolycorine-α-N-oxide, and isocorydine from *L. aurea* exhibited potent cytotoxicity against head and neck squamous cell carcinoma (HNSCC) (Cao et al., 2013; Song et al., 2014). These findings highlight the broad anticancer potential of these compounds.

### 2.4 Anti-inflammatory properties

Pharmacological studies have increasingly demonstrated that alkaloids from *Lycoris* species possess significant anti-inflammatory activities. In particular, lycorine, galanthamine, and narciclasine have been shown to have anti-inflammatory effects through the cholinergic anti-inflammatory pathway (Li et al., 2021). Specifically, lycorine exerts cardioprotective effects through the inhibition of PI3K/AKT and NF-κB-induced inflammatory responses (Tuo et al., 2024), whereas narciclasine functions through the suppression of both the NF-κB and MAPK pathways (Shen et al., 2019). Additionally, 1-hydroxy-ungeremine (137) and N-methoxycarbonyl-2-demethyl-isocorydione (138) isolated from *Lycoris* radiata markedly inhibited COX-2 (90%), indicating their potential for anti-inflammatory drug development (Liu et al., 2015). Furthermore, 2-demethyl-isocorydine (123), dehydrocrebanine(125), and isocorydine (124) extracted from *Lycoris aurea* exhibited anti-inflammatory activity with more than 85% inhibition of cyclooxygenase-2 (COX-2) (Cao et al., 2013).

### 2.5 Neuroprotective effects

*Lycoris* alkaloids exhibit neuroprotective activity. Several neuroprotective alkaloids have been isolated and identified from the bulbs of Lycoris aurea. Among these, 2α-hydroxy-6-O-n-butyloduline (38), O-n-butyllycorenine (50), and N-(chloromethyl)lycoramine (16) demonstrated significant protective effects against both CoCl_2_-induced hypoxic injury and H_2_O_2_-induced oxidative damage. Among the known alkaloids, galantamine specifically targeted CoCl_2_-induced damage, whereas N-demethylgalantamine (9) and lycorinine-type alkaloids were effective in both injury models. The neuroprotective effects of these compounds are achieved mainly by inhibiting neuronal apoptosis and reducing oxidative stress (Jin et al., 2014). Alkaloids isolated from *Lycoris* sp*rengeri* exhibited protective effects against neuronal damage induced by H_2_O_2_ (oxidative damage), CoCl_2_ (hypoxic damage), and Aβ_25-35_ (amyloid toxicity). O-methyllycorenine (52) and hippadine (86) significantly protected against H_2_O_2_-induced damage, with O-methyllycorenine(52) increasing cell viability from 56.1% to 68.9% at 25 μM (p< 0.01). In response to CoCl_2_-induced damage, lycosprenine (87), O-methyllycorenine (52), and tortuosine (89) all exhibited notable activity, with lycosprenine (87) increasing viability to 64.2% at 25 μM (control: 51.6%). Notably, 2α-methoxy-6-O-methyllycorinine (54) showed particularly strong protection against Aβ_25-35_ toxicity, increasing viability to 77.6% at 12.5 μM (control: 59.5%). Structural–activity relationship analysis revealed that methoxylation at the C-2 position significantly enhanced bioactivity. Additionally, the broad effectiveness of lycorinine-type alkaloids across multiple models suggests their potential multitarget mechanisms of action (Wu et al., 2014). Neuroprotective compounds were also isolated from *Lycoris radiata*. In addition to known neuroprotective alkaloids such as galantamine, three compounds, namely, 2α-methoxy-6-O-ethyloduline (54), O-demethyllycoramine N-oxide (14), and N-chloromethyl ugiminoline (66) demonstrated significant protective effects against both H_2_O_2_- and CoCl_2_-induced cellular damage. N-Chloromethylugiminoline exhibited significant protective effects against Aβ_25-35_-induced cellular damage. These findings indicate that neuroprotective alkaloids are widely distributed among *Lycoris* species and that structurally similar alkaloid compounds may have therapeutic potential for related diseases (Li et al., 2013).

### 2.6 Other pharmacological activities

*Lycoris* alkaloids exhibit broad medicinal potential with diverse pharmacological activities. Notably, lycorine (80) shows unique efficacy in treating cutaneous fibrotic disorders, particularly refractory hypertrophic scars. Research has demonstrated that lycorine (80) specifically inhibits hypertrophic scar fibroblast (HSF) proliferation while inducing apoptosis, without affecting normal fibroblasts (NFs). The mechanism involves mainly i the suppression of collagen and α-smooth muscle actin (α-SMA) expression in HSFs, which reduces extracellular matrix (ECM) accumulation and consequently regulates HSF metabolism (Dong et al., 2022). These findings indicate that lycorine may serve as a therapeutic agent for hypertrophic scars because of its proapoptotic and antifibrotic effects. However, further studies are needed to fully understand its mechanisms of action and evaluate its clinical safety. *Lycoris* alkaloids also exhibit antimalarial activities. While lycorenine (55) and haemanthamine (29) have demonstrate weaker effects than artemisinin dose, they specifically target key enzymes in the plasmodial FAS-II biosynthesis pathway (Nair and van Staden, 2019). Their structural complexity provides a valuable basis for developing novel antimalarial agents (Narula et al., 2019). These alkaloids also demonstrate antifungal activities. Studies have shown that methanol extracts from *Lycoris radiata* significantly inhibit *Magnaporthe oryzae* mycelial growth, with lycorine (80) and narciclasine (100) likely serving as the main active components (Qiao et al., 2023). Lycorine (80) has been shown to mitigate carbon tetrachloride (CCl_4_)-induced hepatic fibrosis by modulating the JAK2/STAT3 and PI3K/AKT signaling pathways, suggesting its potential therapeutic application for liver injury-related diseases (Y. Tang et al., 2024).

Among the diverse alkaloids identified in *Lycoris radiata*, galanthamine (13) has demonstrated the most prominent clinical value. As a reversible acetylcholinesterase (AChE) inhibitor and nicotinic acetylcholine receptor modulator, this compound has been approved by the U.S. Food and Drug Administration (FDA) for the clinical treatment of AD (Howes and Houghton, 2003). In contrast, lycorine (80) has a broad exhibits a broader spectrum of pharmacological activities, including antiviral, antitumor, analgesic, hepatoprotective, and anti-inflammatory effects; however, its clinical application remains limited because of its significant cytotoxicity (Su et al., 2023; Zhao et al., 2023). Another alkaloid, lycoramine (18), also possesses AChE inhibitory activity, albeit with lower potency than galanthamine (13) does (Ding et al., 2017; Nair and Van Staden, 2021). Moreover, narciclasine (134) primarily exerts anticancer effects through the induction of apoptosis (Li et al., 2021). In summary, while the use of galanthamine (13) has been well-established for the treatment of neurological disorders, other *Lycoris* alkaloids show promising potential in oncology and infectious disease management. However, their therapeutic application is currently limited by toxicity concerns, necessitating further structural modifications and research to enhance safety profiles and clinical applicability.

## 3 Regulatory mechanism and bioengineering of active compounds

The limited availability of Amaryllidaceae alkaloids (AAs) from natural sources presents significant challenges, including their low abundances in plants, high extraction costs, and fragile plant sources with poor regenerative capacities. Furthermore, unsuccessful cultivation attempts and costly isolation techniques further restrict their supply (Sanyal et al., 2023). These factors collectively hinder efforts to meet the growing demand for these valuable medicinal compounds (Shi et al., 2025).

Although significant progress has been made in elucidating key biosynthetic pathways (Desgagné-Penix, 2021), the complete biosynthesis of *Lycoris* alkaloids remains unclear. Given their limited availability and costly extraction, alternative synthesis methods are being actively explored. Current approaches include biomimetic oxidation and intramolecular Heck reactions using phenol derivatives. Further investigation of *Lycoris* alkaloid biosynthetic pathways and their regulatory mechanisms could enable sustainable production and facilitate new therapeutic applications.

### 3.1 Overview of biosynthesis pathways

Alkaloids are valuable secondary metabolites that are widely present in bacteria, fungi, and plants. These compounds not only function in organismal growth (Lam et al., 2022), but also exhibit various biological activities (Kishimoto et al., 2016), contributing significantly to human health. However, the current understanding of their biosynthetic pathways in plants remains limited, particularly in *Lycoris radiata*, a species renowned for its therapeutic potential against AD. *Allium sativum* alkaloids, particularly those known for their therapeutic effects against AD, undergo a multistep biosynthetic process involving methylation, reduction, oxidation, condensation, hydroxylation, phenol–phenol coupling, and oxygen bridge formation (Kilgore et al., 2014).

**Figure 5:**
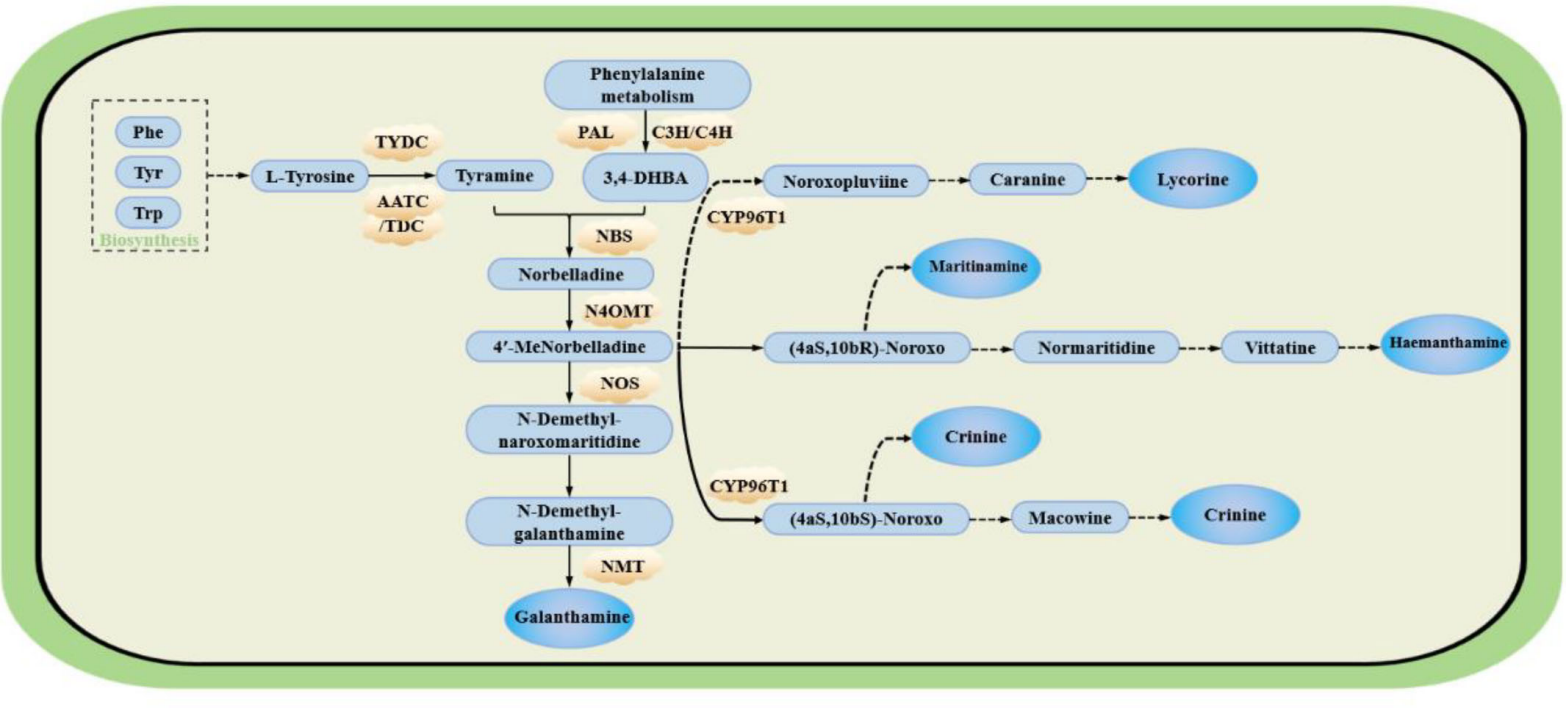
The possible pathway of main alkaloid synthesis in Lycoraceae [based on (Li et al., 2019) (Hotchandani et al., 2019) (Li et al., 2022)] Phe, phenylalanine; Tyr, tyrosine; Trp, tryptophan; PAL, phenylalanine ammonia lyase; C4H, cinnamate-4-hydroxylase; C3H, cinnamate-3-hydroxylase; TYDC, tyrosine decarboxylase; NBS, norbelladine synthase; CYP96T1, cytochrome P450 family enzyme; AADC, aromatic-L-amino-acid decarboxylase; TDC, L-tryptophan decarboxylase; N4OMT, norbelladine O-methyltransferase; NOS, Noroxomaritidine synthase; NMT, S-adenosyl-L-homocysteine:norgalanthamine N-methyltransferase; 3,4-DHBA, 3,4-Dihydroxybenzaldehyde; 4′-MeNorbelladine, 4’ Methylnorbelladine; (4aS,10bR)-Noroxo, (4aS,10bR)-Noroxomaritidine; (4aS,10bS)-Noroxo, (4aS,10bS)-Noroxomaritidine; Macowine, (4aR,10bS)-Normaritidine. PAL, C3H, C4H, TYDC, CYP96T1, N4OMT, NBS, and NMT have all been studied in Lycoris.

Two amino acids, L-phenylalanine and L-tyrosine, serve as the primary precursors for alkaloid biosynthesis in *Allium sativum*. Phenylalanine is first converted to trans-cinnamic acid by phenylalanine ammonia-lyase (PAL). Trans-cinnamic acid subsequently undergoes cytochrome P450-mediated two-step hydroxylation to yield 3,4-dihydroxybenzaldehyde (3,4-DHBA). In parallel, tyrosine decarboxylation is catalysed by tyrosine decarboxylase (TYDC) to produce tyramine. This tyramine then condenses with 3,4-DHBA through the action of norbelladine synthase (NBS) to form norbelladine. Norbelladine is converted to 4′-O-methylnorbelladine by O-methyltransferase (OMT). As a central precursor in the *Lycoris* alkaloid biosynthesis pathway, 4′-O-methylnorbelladine undergoes cytochrome P450-mediated oxidative C-C phenol coupling reactions. Product analysis revealed three major alkaloid types derived from this intermediate: lycorine-type, galantamine-type, and narciclasine-type alkaloids, as illustrated in **Figure 5** (Qingzhu Li et al., 2022).

### 3.2 Functions and regulation of key enzymes

Studies have demonstrated that alkaloid biosynthesis is regulated by multiple factors through complex physiological processes. The identification of norbelladine 4′-O-methyltransferase (N4′OMT) enables future characterization of related enzymes in the Amaryllidaceae alkaloid biosynthetic pathway. Genes coexpressed with NpN4′OMT may serve as candidate genes for subsequent steps in this pathway, particularly given role of N4′OMT in dinucleotide cofactor (NADPH/NADH) binding (Kavanagh et al., 2008; Porté et al., 2013). 2-oxoglutarate-dependent dioxygenases and cytochrome P450 enzymes exhibit hydroxylation activities, representing promising candidate gene families for hydroxylation steps in Amaryllidaceae (Lester et al., 1997; Nelson and Werck-Reichhart, 2011). Transcriptomic and metabolomic analyses of *Lycoris radiata* were conducted to investigate galantamine biosynthesis across different tissues. The results demonstrated that *LrNNR*, *LrN4OMT*, and *LrCYP96T* exhibited the highest expression levels in bulbs, which correlated with the significantly greater galantamine accumulation observed in bulbs than in roots and leaves (Tang et al., 2023). OMT was identified as the key enzyme mediating substrate- and region-specific polymethylation in galantamine biosynthesis, with additional roles in alkaloid metabolism (Chang et al., 2015; Hawkins and Smolke, 2008). NMT, another methyltransferase, has been demonstrated to catalyse phenylpropanoid alkaloid biosynthesis (Grobe et al., 2011).

SWATH-MS-based quantitative proteomic analysis revealed 720 significantly differentially expressed proteins in *L. longituba* with high alkaloid contents. These proteins were predominantly enriched in biosynthetic pathways, particularly those involved in amino acid metabolism and starch/sucrose metabolism. Notably, O-methyltransferase (OMT) and N-methyltransferase (NMT) participate directly in the galanthamine biosynthetic pathway (Tang et al., 2023). Integrated transcriptomic and metabolomic profiling of *Lycoris radiata* revealed eight key genes implicated that are involved in galanthamine biosynthesis (including *LrPAL2*, *LrC4H2*, and *LrN4OMT*). Among these genes, *LrNNR* and *LrN4OMT* displayed the greatest expression levels in bulb tissues, which concurrently the greatest increase in galanthamine accumulation (0.75 mg/g dry weight) - 1.42-fold and 2.78-fold greater than that in roots and leaves, respectively. These findings strongly support the pivotal role of *LrNNR* and *LrN4OMT* in galanthamine production (Park et al., 2019). Furthermore, comprehensive functional characterization of three NADPH-cytochrome P450 reductases (e.g., *LrCPR1*, *LrCPR2*, and *LrCPR3*) in *L. radiata* was performed. *In vitro* enzymatic analyses coupled with heterologous yeast expression systems confirmed their capacity to facilitate CYP96T6 activity (a P450 enzyme mediating galanthamine precursor biosynthesis), resulting in significantly elevated yields of N-demethylnarwedine, a crucial biosynthetic intermediate (Wu et al., 2024). Complementary work involving *LrOMT* cloning and heterologous expression in *E. coli* established its bifunctional O-methylation capability, offering mechanistic understanding of the structural diversification of Amaryllidaceae alkaloids (Li et al., 2019).

### 3.3 Functions and regulation of key enzymes

Plants have evolved diverse defense mechanisms to adapt to various natural environments. In Hymenocallis, light quality significantly influences alkaloid accumulations. Specifically, red light decreases GAL, LYC, and LYCM levels, whereas blue light increases their accumulation. Notably, blue light provides optimal conditions for inducing alkaloid production in this species. At the genetic level, blue light treatment significantly altered gene transcription patterns in *Hymenocallis*, potentially promoting alkaloid accumulation. Key biosynthetic genes including *PAL*, *C4H*, and *TYDC* were notably upregulated under blue light conditions (Qingzhu Li et al., 2022). Compared with fully irrigated controls, drought stress induced a steady increase in coptisine levels. However, unlike other environmental stresses, drought was the only condition that significantly elevated coptisine accumulation in most plants. Notably, under severe drought conditions (Yahyazadeh et al., 2018), t coptisine levels plateaued, suggesting a stress threshold effect that requires further investigation. Additionally, soil physicochemical properties influence coptisine production (Frick et al., 2017). Soil physicochemical properties significantly influence lupin growth. As the soil pH increases, both the QA levels and the seed yields decrease in lupin plants, along with a reduction in protein content (Rodés-Bachs and Van der Fels-Klerx, 2023).

### 3.4 Impact of alternative splicing on alkaloid synthesis

The pharmacological potential of *Lycoris* alkaloids has generated significant interest in medical research. However, their biosynthetic pathways remain incompletely understood. These complex metabolic processes are tightly regulated across multiple levels—including transcriptional, translational, and post-translational modifications—to adapt to various developmental stages and environmental changes (Lam et al., 2022). Elucidating alkaloid biosynthesis mechanisms is crucial for understanding the secondary metabolism of Amaryllidaceae and facilitating their sustainable development. Alternative splicing represents a key regulatory mechanism within the complex network controlling plant alkaloid biosynthesis.

Alternative splicing, a key gene regulatory mechanism, controls post-transcriptional mRNA processing (Laloum et al., 2018). This process enables cells to produce multiple protein variants from a limited number of genes (Baralle and Giudice, 2017). The biosynthesis of alkaloids in *Lycoris* species involves a unique plant metabolic pathway in which developmental stage- and stress-responsive alternative splicing regulates multiple genes (Chang et al., 2015). A study by Carqueijeiro of *Catharanthus roseus* identified alternative splicing events in a monoterpenoid indole alkaloid (MIA) biosynthesis-related gene, revealing its regulatory roles in both MIA production and plant defense responses (Carqueijeiro et al., 2021). In *Ginkgo biloba*, alternative splicing was shown to regulate flavonoid and terpenoid biosynthesis pathways (He et al., 2022). This mechanism also plays a crucial role in stress response regulation in woody plants (Chen et al., 2020), potentially providing evolutionary advantages for environmental adaptation (Liu et al., 2022).

### 3.5 Impact of alternative splicing on alkaloid synthesis

Synthetic biology plays a key role in alkaloid research by elucidating the biosynthetic pathways and regulatory mechanisms of alkaloids in plants. This approach helps reveal their essential functions and potential applications in plant physiological and ecological processes.

Since the synthesis of secondary metabolite alkaloids in plants is inherently limited and yields are low, synthetic biology methods are needed to produce the desired components. The first step in this approach is to select an appropriate host organism (Koirala et al., 2022). After the host is determined, the metabolism of precursors for synthesizing target components can be regulated through gene modulation. These include three main strategies—deleting genes, replacing endogenous enzymes with more active homologues, and overexpressing endogenous metabolic genes—all aimed at regulating the synthesis pathway to achieve higher yields. Gene deletion, replacement of endogenous enzymes with more active homologues, and overexpression of endogenous metabolic genes are three approaches to regulate the metabolism of the host organism. These methods can be used to redirect the synthesis pathways of specific target metabolites, thereby increasing the production of desired compounds. The pathway for a particular target metabolite can be determined using these strategies. The entire process requires step-by-step analysis of the target compounds’ intermediates on the basis of the host’s metabolic regulation. This analysis forms the complete synthetic pathway for the target compound. Ultimately, specific enzymes are screened to carry out each reaction in this pathway, achieving the biosynthesis of the desired products (Ausländer et al., 2017; Kitney and Freemont, 2012).

Compared with other drugs, galanthamine, a reversible acetylcholinesterase inhibitor, is relatively less toxic to humans. It is currently the only approved treatment for early-stage AD (Houghton et al., 2006). To date, the chemical synthesis of galanthamine has been explored (Marco-Contelles et al., 2006). However, owing to its complex structure and the low yield of multistep chemical synthesis, the production of galantamine through biosynthesis remains economically challenging. Consequently, plant extraction remains the predominant source of galantamine for medical applications (Takos and Rook, 2013).

## 4 Conclusion and perspectives

*Lycoris* species, particularly *Lycoris radiata*, are rich in structurally diverse Amaryllidaceae alkaloids that display a wide range of pharmacological activities, including antitumor, neuroprotective, antiviral, and anti-inflammatory effects. Among these compounds, galanthamine and lycorine have shown great therapeutic potential, especially in the treatment of AD and various cancers. Despite their medicinal value, the development and utilization of these compounds face multiple limitations that require urgent resolution. One of the most critical challenges is the low natural abundances of key alkaloids. For instance, the extraction of a small amount of galanthamine necessitates several tons of wild *Lycoris* biomass, leading to serious overharvesting, habitat destruction, and the depletion of wild germplasm resources. This situation highlights the urgent need for strategies that ensure sustainable use, including the conservation of germplasm, ex situ cultivation, and strict regulation of wild collection practices.

In addition to resource constraints, the biosynthetic pathways of many Lycoris alkaloids remain poorly characterized. Although several enzymes such as N4OMT, CYP96T1, and NMT have been identified, the downstream steps and regulatory networks that are involved in alkaloid synthesis are still largely unknown. This lack of comprehensive understanding severely limits the application of synthetic biology and metabolic engineering approaches aimed at improving yields. Furthermore, the spatial and temporal distribution of alkaloid biosynthesis in different tissues and developmental stages has been insufficiently explored, which complicates targeted manipulation strategies.

Biotechnological approaches such as tissue cultures and *in vitro* production systems offer promising alternatives, but they are still limited by problems such as browning of tissues, low regeneration efficiency, poor accumulation of target metabolites, and high production costs. These technical bottlenecks have prevented large-scale industrial application. Moreover, the pharmacological effects of single compounds versus complex alkaloid mixtures remain poorly understood, and the lack of data on synergistic or antagonistic interactions poses challenges for clinical development and safety assessment.

Current research focuses primarily on the application of galantamine in AD treatment, as it represents the most therapeutically promising alkaloid in *Lycoris* species. However, the structural complexity of galantamine makes its complete chemical synthesis currently unfeasible. The biosynthetic pathway of galantamine involves five main key steps: precursor synthesis, methylation, phenolic oxidative coupling, reduction and cyclization, and transport and accumulation(**Figure 5**).

In addition to increasing *Lycoris* yields, since the alkaloid contents in *Lycoris* are relatively low, a considerable number of current studies have focused on increasing alkaloid yields. Many studies have shown that there is a complex interaction between endophytes in *Lycoris* and the synthesis of host alkaloids. Inoculation of *Lycoris* endophytes can increase the alkaloid contents, and different endophytes are associated with the synthesis of different alkaloids. However, relatively few studies have investigated the types of endophytes and the types of *Lycoris* inoculated, and further in-depth research is needed. Most existing studies are still at the laboratory stage and lack feasibility studies on large-scale inoculation. In addition to adding inducers, which is an effective method to increase the alkaloid contents in *Lycoris*, methyl jasmonate is one of them (Wang et al., 2016). To date, several key genes involved in the lycorine alkaloid biosynthesis pathway have been identified. Nevertheless, environmental factors play an equally crucial role in regulating the expression of these genes. Jasmonate ZIM-domain (JAZ) proteins, as core regulators of the jasmonic acid (JA) signaling pathway, participate in multiple biological processes including secondary metabolism (particularly alkaloid biosynthesis), stress responses, and plant growth and development. Previous studies have demonstrated that environmental stimuli such as drought and light exposure can activate alkaloid biosynthetic genes (e.g., TYDC and N4OMT) through the ABA and JA signaling pathways (Sha et al., 2024; Chen et al., 2023). Furthermore, experimental evidence confirms that exogenous JA treatment significantly enhances the production of lycorine-type alkaloids in Amaryllidaceae species (Wang et al., 2019). On the basis of the current research status, compared with simply expanding the planting scale, shifting the research focus to cultivating *Lycoris* varieties with high alkaloid contents or constructing efficient prokaryotic/eukaryotic overexpression systems has become a more effective strategy to solve the alkaloid production bottleneck for the *Lycoris* genus. Future research should establish a core germplasm resource bank for multiple species, develop synthetic microbial community inoculation technology, and construct a CRISPR-Cas9-mediated metabolic pathway editing system. Moreover, improving basic research such as endophyte isolation rate data, standardized methyl jasmonate (MeJA) treatment protocols, and optimization of heterologous expression systems, is necessary.

## Ethics statement

Informed consent was obtained from all subjects involved in the study.
