# Supplementary material for: Medicinal compounds and biotechnology of Amaryllidaceae alkaloids in Lycoris radiata
Source: Front Plant Sci. 2025 Dec 15;16:1639654. doi: 10.3389/fpls.2025.1639654 (PMC12745432; doi:10.3389/fpls.2025.1639654)
Supplement: Supplementary file 1 [file Table1.docx]

**Table 1**. Alkaloids Identified in Lycoris Species: Structural Types, Plant Sources, and Pharmacological Activities

| Name | Number | Structural type | Plant source | Tissue distribution | Pharmacological activity | References |
| --- | --- | --- | --- | --- | --- | --- |
| 2R-Hydroxy-N,O-dimethylnorbelladine | 1 | Belladine-type | *Lycoris squamigera*. | Bulbs | NA | (Kitajima et al., 2009) |
| Amabiline | 2 | Crinine-type | *Lycoris radiata* | Bubls | Aphicial activit | (Yan et al., 2018) |
| Ambelline | 3 | Crinine-type | *L. aurea*  *L. radiata* | Bulbs | NA | (Yongqiang Tian, Chunyun Zhang, & Mingquan Guo, 2015a) |
| Crinine | 4 | Crinine-type | *Lycoris incarnata*. | Bulbs | NA | (H. Y. Li, Ma, Xu, & Hong, 1987) |
| Crinamabine | 5 | Crinine-type | *L. radiata*  *L. radiata* | Bulbs | NA | (Yongqiang Tian, Chunyun Zhang, & Mingquan Guo, 2015b) |
| Crinamidine | 6 | Crinine-type | *L. aurea*  *L. guangxiensis*  *L. radiata* | Bulbs | NA | (Yongqiang Tian et al., 2015b) |
| 1,2,11,12-Tetradehydrogalanthamine * | 7 | Galanthamine-type | *L. radiata* | Bulbs | NA | (Yongqiang Tian et al., 2015b) |
| 11β-Hydroxygalanthamine | 8 | Galanthamine-type | *L. longituba*  *L. radiata* | Bulbs | AChE inhibitory activity | (Zhu et al., 2015)  (Yan et al., 2018) |
| N-Norgalanthamine/N-Demethylgalanthamine | 9 | Galanthamine-type | *L. aurea*  *L. guangxiensis*  *L. longituba*  *L. radiata* | Bulbs | AChE inhibitory activity；Neuroprotective effects | (Zhu et al., 2015)  (Xi et al., 2009)  (An Jin et al., 2014)  (H. Y. Li et al., 1987) |
| N-Allylnorgalanthamine | 10 | Galanthamine-type | *L. radiata*  *L. guangxiensis* | Bulbs | Aphicial activity | (H. Y. Li et al., 1987)  (Yan et al., 2018) |
| Galanthamine N-oxide | 11 | Galanthamine-type | *L. incarnata*  *L. radiata* | Flowers,  Bulbs | NA | (Kihara et al., 1994)  (X. Li et al., 2013)  (Kihara, Konishi, Xu, & Kobayashi, 1991) |
| N-(Chloromethyl)galanthamine | 12 | Galanthamine-type | *L. aurea*  *L. longituba* | Bulbs | AChE inhibitory activity；Neuroprotective effects | (An Jin et al., 2014)  (Zhu et al., 2015) |
| Galanthamine | 13 | Galanthamine-type | *L. albiflora*  *L. aurea*  *L. chinensis*  *L. guangxiensis*  *L. haywardii*  *L. incarnata*  *L. longituba*  *L. radiata*  *L. radiata var. pumila*  *L. sprengeri*  *L. squamigera*  *L. traubii* | Bulbs,  Seeds  ,Leaves,  Root hair | AChE inhibitory activity；Neuroprotective effects；Aphicial activit; Myasthenia gravis；Anti-inflammatory activity | (Maki Jitsuno et al., 2011)  (Y. Guo et al., 2014) |
| O-Demethyllycoramine-N-oxide | 14 | Galanthamine-type | *L. radiata* | Bulbs | NA | (X. Li et al., 2013) |
| O-Demethyllycoramine | 15 | Galanthamine-type | *L. incarnata*  *L. longituba*  *L. radiata*  *L. squamigera* | Flower,  bulbs | AChE inhibitory activity | (Verpoorte & Schripsema, 1994)  (Y. Guo et al., 2014)  (X. Li et al., 2013)  (Kihara et al., 1991) |
| N-(Chloromethyl)lycoramine | 16 | Galanthamine-type | *L. aurea*  *L. longituba* | Bulbs | AChE inhibitory activity；Neuroprotective effects | (Zhu et al., 2015)  (An Jin et al., 2014) |
| Lycoramine-N-oxide | 17 | Galanthamine-type | *L. albiflora*  *L. aurea*  *L. radiata* | Seeds,  leaves,  root hair | NA | (Yongqiang Tian et al., 2015b)  (X. Li et al., 2013)  (Kihara et al., 1991)  (Maki Jitsuno et al., 2011) |
| Lycoramine | 18 | Galanthamine-type | *L. albiflora*  *L. aurea*  *L. chinensis*  *L. guangxiensis*  *L. haywardii*  *L. incarnata*  *L. longituba*  *L. radiata*  *L. radiata var. pumila*  *L. sprengeri*  *L. squamigera*  *L. traubii* | Bulbs | Myasthenia gravis；Post-polio syndrome | (Maki Jitsuno et al., 2011)  (Y. Guo et al., 2014)  (Mu et al., 2010)  (H. Y. Li et al., 1987) |
| Norlycoramine | 19 | Galanthamine-type | *L. albiflora*  *L. chinensis*  *L. incarnata*  *L. longituba*  *L. radiata*  *L. sprengeri*  *L. squamigera* | Bulbs | NA | (Y. Guo et al., 2014) |
| Narwedine | 20 | Galanthamine-type | *L. albiflora*  *L. aurea*  *L. guangxiensis*  *L. longituba*  *L. squamigera* | Bulbs | NA | (Y. Guo et al., 2014)  (Yongqiang Tian et al., 2015b)  (H. Y. Li et al., 1987) |
| Sanguinine | 21 | Galanthamine-type | *L. aurea*  *L. chinensis*  *L. incarnata*  *L. longituba*  *L. radiata*  *L. squamigera* | Flowers; bulbs | AChE inhibitory activity | (Kihara et al., 1994)  (Y. Guo et al., 2014) |
| Lycosinine B | 22 | Galanthindole-type | *L. sprengeri* | Bulbs | NA | (W. M. Wu et al., 2014) |
| 3α-Hydroxy-6β-acetylbulbispermine | 23 | Haemanthamine-type | *L. radiata* | Bulbs | Antitumor activity；Antimalarial activity | (B. Hao, S.-F. Shen, & Q.-J. Zhao, 2013) |
| 3α-Methoxy-6β-acetylbulbispermine | 24 | Haemanthamine-type | *L. radiata* | Bulbs | In vitro antimalarial activity；Antitumor activity Antimalarial activity； | (Bin Hao et al., 2013) |
| 3α,6β-Diacetylbulbispermine | 25 | Galanthindole-type | *L. radiata* | Bulbs | In vitro antimalarial activity；Antitumor activity；Antimalarial activity | (Bin Hao et al., 2013) |
| 6β-Acetyl-8-hydroxy-9-methoxycrinamine | 26 | Haemanthamine-type | *L. radiata* | Bulbs | Antitumor activity | (Z. M. Liu et al., 2015) |
| 6-Hydroxycrinamine | 27 | Haemanthamine-type | *L. radiata* | Bulbs | NA | (Feng et al., 2011) |
| 6β-Acetoxycrinamine | 28 | Haemanthamine-type | *L. radiata* | Bulbs | Antitumor activity | (Z. M. Liu et al., 2015)  (Feng et al., 2011) |
| Haemanthamine | 29 | Haemanthamine-type | *L. albiflora*  *L. aurea*  *L. chinensis*  *L. incarnata*  *L. radiata*  *L. sprengeri*  *L. squamigera* | Bulbs | NA | (Maki Jitsuno et al., 2011)  (Y. Guo et al., 2014)  (Yongqiang Tian et al., 2015b) |
| Haemanthidine | 30 | Haemanthamine-type | *L. albiflora*  *L. incarnata*  *L. longituba*  *L. radiata*  *L. sprengeri*  *L. squamigera* | Bulbs | AChE inhibitory activity | (Maki Jitsuno et al., 2011)  (Y. Guo et al., 2014)  (Kihara et al., 1991) |
| 8-O-Demethylmaritidine | 31 | Haemanthamine-type | *L. radiata* | Bulbs | NA | (Yongqiang Tian et al., 2015b)  (L. Wang et al., 2010) |
| 11-Hydroxyvittatine-N-oxide | 32 | Haemanthamine-type | *L. aurea* | Bulbs | NA | (Yongqiang Tian et al., 2015b) |
| 11-Hydroxyvittatine | 33 | Haemanthamine-type | *L. radiata*  *L. squamigera* | Bulbs | NA | (L. Wang et al., 2010) |
| Vittatine | 34 | Haemanthamine-type | *L. aurea*  *L. guangxiensis*  *L. radiata* | Bulbs  Flowers | NA | (Yongqiang Tian et al., 2015b)  (Kihara et al., 1991)  (An Jin et al., 2014) |
| 2α-Methoxy-6-O-ethyloduline | 35 | Homolycorine-type | *L. radiata* | Bulbs | Antiviral activities | (S. D. Huang et al., 2013)  (X. Li et al., 2013) |
| 2α-Methoxy-6-O-methyloduline | 36 | Homolycorine-type | *L. aurea*  *L. guangxiensis*  *L. radiata* | Bulbs | Antiviral activities | (Na Liao, Mingzhang Ao, Peng Zhang, & Longjiang Yu, 2012)  (Yongqiang Tian et al., 2015b)  (S. D. Huang et al., 2013) |
| 2α-Hydroxy-6-O-methyloduline | 37 | Homolycorine-type | *L. albiflora*  *L. aurea*  *L. radiata* | Bulbs | Neuroprotective effects | (Na Liao et al., 2012)  (Yongqiang Tian et al., 2015b)  (X. Li et al., 2013) |
| 2α-Hydroxy-6-O-n-butyloduline | 38 | Homolycorine-type | *L. aurea* | Bulbs | Neuroprotective effects | (An Jin et al., 2014)  (Na Liao et al., 2012) |
| 2α-Hydroxyoduline | 39 | Homolycorine-type | *L. aurea* | Bulbs | NA | (Na Liao et al., 2012)  (Yongqiang Tian et al., 2015b) |
| Oduline | 40 | Homolycorine-type | *L. aurea*  *L. guangxiensis*  *L. radiata* | Bulbs | NA | (Z. M. Liu et al., 2015) |
| 2*α*-Hydroxy-8-*O*-demethylhomolycorine-*N*-oxide | 41 | Homolycorine-type | *L. radiata* | Bulbs | NA | (Z. M. Liu et al., 2015)  (J.-H. Song, L. Zhang, & Y. Song, 2014) |
| 8-demethyl-homolycorine-*α*-*N*-oxide | 42 | Homolycorine-type | *L. radiata* | Bulbs | Antitumor activity | (Z. M. Liu et al., 2015)  (J.-H. Song et al., 2014) |
| 8-O-Demethylhomolycorine | 43 | Homolycorine-type | *L. haywardii*  *L. radiata*  *L. radiata var. pumila* | Bulbs  Flowers | NA | (Y. Guo et al., 2014) |
| 9-O-Demethylhomolycorine | 44 | Homolycorine-type | *L. albiflora*  *L. radiata* | Bulbs | NA | (Maki Jitsuno et al., 2011)  (X. Li et al., 2013) |
| 9-O-Demethyl-2α-hydroxyhomolycorine | 45 | Homolycorine-type | *L. radiata* | Bulbs | NA | (X. Li et al., 2013) |
| 8,9-Methylenedioxyhomolycorine-N-oxide | 46 | Homolycorine-type | *L. radiata* | Bulbs | Antitumor activity；Antimalarial activity | (Yongqiang Tian et al., 2015b)  (Bin Hao et al., 2013) |
| 8-O-Acetylhomolycorine-N-oxide | 47 | Homolycorine-type | *L. radiata* | Bulbs | Antitumor activity；Antimalarial activity | (Feng et al., 2011) |
| Homolycorine-N-oxide | 48 | Homolycorine-type | *L. albiflora*  *L. radiata* | Bulbs  Flowers | In vitro antimalarial activity；Antitumor activity | (Bin Hao et al., 2013)  (Maki Jitsuno et al., 2011) |
| Homolycorine | 49 | Homolycorine-type | *L. albiflora*  *L. aurea*  *L. haywardii*  *L. radiata*  *L. radiata var. pumila*  *L. sprengeri* | Flowers  Bulbs | NA | (Maki Jitsuno et al., 2011)  (Y. Guo et al., 2014)  (W. M. Wu et al., 2014) |
| O-n-butyllycorenine | 50 | Homolycorine-type | *L. aurea* | Bulbs | Neuroprotective effects | (An Jin et al., 2014) |
| O-Ethyllycorenine | 51 | Homolycorine-type | *L. radiata* | Bulbs | NA | (X. Li et al., 2013) |
| O-Methyllycorenine | 52 | Homolycorine-type | *L. aurea*  *L. radiata*  *L. sprengeri* | Bulbs  Flowers | Neuroprotective effects | (An Jin et al., 2014)  (X. Li et al., 2013)  (W. M. Wu et al., 2014) |
| O-Methyllycorenine-N-oxide | 53 | Homolycorine-type | *L. radiata* | Flowers | NA | (Kihara et al., 1991) |
| 2α-Methoxy-6-O-methyllycorenine | 54 | Homolycorine-type | *L. sprengeri* | Bulbs | NA | (W. M. Wu et al., 2014) |
| Lycorenine | 55 | Homolycorine-type | *L. albiflora*  *L. radiata*  *L. sprengeri* | Bulbs | NA | (W. M. Wu et al., 2014)  (Boit, Döpke, & Stender, 1958)  (S.-D. HUANG et al., 2013) |
| Radiatine | 56 | Homolycorine-type | *L. radiata* | Bulbs | NA | (X. Li et al., 2013) |
| Hippeastrine | 57 | Homolycorine-type | *L. albiflora*  *L. aurea*  *L. radiata* | Bulbs  Flowers | Antitumor activity；Antiviral activities | (X. Li et al., 2013)  (Maki Jitsuno et al., 2011)  (An Jin et al., 2014) |
| Hippeastrine-N-oxide | 58 | Homolycorine-type | *L. albiflora*  *L. radiata* | Bulbs  flowers | NA | (Maki Jitsuno et al., 2011; Kihara et al., 1991) |
| Unsevine | 59 | Homolycorine-type | *L. radiata* | Bulbs | NA | (Yongqiang Tian et al., 2015b) |
| Hostasinine A | 60 | Hostasinine-type | *L. albiflora* | Bulbs | NA | (Maki Jitsuno et al., 2011) |
| Ismine | 61 | Ismine-type | *L. squamigera* | Bulbs | NA | (Kitajima et al., 2009) |
| Assoanine | 62 | Lycorine-type | *L. albiflora*  *L. longituba* | Bulbs | NA | (Y. Guo et al., 2014) |
| Caranine | 63 | Lycorine-type | *L. albiflora*  *L. aurea*  *L. chinensis*  *L. longituba*  *L. radiata*  *L. radiata var. pumila*  *L. sprengeri*  *L. squamigera* | Bulbs | NA | (Y. Guo et al., 2014) |
| Galanthine | 64 | Lycorine-type | *L. albiflora*  *L. aurea*  *L. chinensis*  *L. haywardii*  *L. incarnata*  *L. longituba*  *L. radiata*  *L. sprengeri*  *L. squamigera* | Bulbs  Flowers | NA | (Y. Guo et al., 2014)  (Kihara et al., 1994)  (L. Wang et al., 2010)  (W. M. Wu et al., 2014) |
| Incartine | 65 | Lycorine-type | *L. albiflora*  *L. aurea*  *L. chinensis*  *L. haywardii*  *L. incarnata*  *L. longituba*  *L. sprengeri*  *L. squamigera* | Bulbs  Flowers | AChE inhibitory activity | (Y. Guo et al., 2014)  (Kihara et al., 1994)  (Zhu et al., 2015) |
| N-(Chloromethyl)ungiminorine | 66 | Lycorine-type | *L. radiata* | Bulbs | NA | (X. Li et al., 2013) |
| N-(Chloromethyl)narcissidine | 67 | Lycorine-type | *L. longituba*  *L. sprengeri* | Bulbs | AChE inhibitory activity | (Zhu et al., 2015)  (W. M. Wu et al., 2014) |
| Narcissidine | 68 | Lycorine-type | *L. sprengeri* | Bulbs | NA | (W. M. Wu et al., 2014) |
| (−)-epi-Zephyranthine | 69 | Lycorine-type | *L. radiata* | Bulbs | NA | (X. Li et al., 2013) |
| Anhydrolycorine | 70 | Lycorine-type | *L. albiflora*  *L. aurea*  *L. chinensis*  *L. haywardii*  *L. incarnata*  *L. longituba*  *L. radiata var. pumila*  *L. sprengeri* | Bulbs | NA | (Y. Guo et al., 2014) |
| Dihydrolycorine | 71 | Lycorine-type | *L. aurea*  *L. guangxiensis*  *L. radiata* | Bulbs | NA | (Yongqiang Tian et al., 2015b)  (X. Li et al., 2013) |
| 11-Methoxylycorine | 72 | Lycorine-type | *L. radiata* | Bulbs | NA | (X. Li et al., 2013) |
| Pseudolycorine | 73 | Lycorine-type | *L. guangxiensis*  *L. radiata*  *L. squamigera* | Bulbs | NA | (X. Li et al., 2013)  (H. Y. Li et al., 1987)  (Kitajima et al., 2009) |
| 5,6-Dehydrodihydrolycorine | 74 | Lycorine-type | *L. albiflora*  *L. radiata* | Bulbs | NA | (Y. Guo et al., 2014)  (Feng et al., 2011) |
| 5,6-Dehydrolycorine | 75 | Lycorine-type | *L. radiata* | Bulbs | In vitro antimalarial activity；Antitumor activity；Antimalarial activity | (Yongqiang Tian et al., 2015b)  (Bin Hao et al., 2013) |
| 11,12-Didehydroanhydrolycorine | 76 | Lycorine-type | *L. aurea*  *L. chinensis*  *L. haywardii*  *L. incarnata*  *L. longituba*  *L. radiata*  *L. radiata var. pumila*  *L. sprengeri*  *L. squamigera* | Bulbs | NA | (Y. Guo et al., 2014) |
| 3-hydroxy-anhydrolycorine-N-oxide | 77 | Lycorine-type | *L. caldwelii* | Bulbs | Antitumor Activity； | (Peng Cao et al., 2013) |
| 1-O-(3′-Hydroxybutanoyl)lycorine | 78 | Lycorine-type | *L. traubii* | Bulbs | NA | (Toriizuka et al., 2008) |
| 6-Oxodihydrolycorine | 79 | Lycorine-type | *L. radiata* | Bulbs | Antitumor Activity | (Feng et al., 2011) |
| Lycorine | 80 | Lycorine-type | *L. albiflora*  *L. aurea*  *L. chinensis*  *L. guangxiensis*  *L. haywardii*  *L. incarnata*  *L. longituba*  *L. radiata*  *L. radiata var. pumila*  *L. sprengeri*  *L. squamigera*  *L. traubii* | Bulbs,  Flowers,seeds,leaves,toot-hairs | Antitumor Activity；AChE Inhibitory activity；Aphicial activit; Antiviral Activity；Anti-inflammatory activity | (Maki Jitsuno et al., 2011)  (Y. Guo et al., 2014)  (Kihara et al., 1994) |
| 1,2-Dihydroxy-anhydrolycorine-N-oxide | 81 | Lycorine-type | *L. aurea* | Bulbs | Antitumor activity | (J.-H. Song et al., 2014) |
| anhydrolycorin-7-one | 82 | Lycorine-type | *L. aurea* | Bulbs | NA | (J.-H. Song et al., 2014) |
| 1-hydroxy-anhydrolycorin-7-one | 83 | Lycorine-type | *L. aurea* | Bulbs | NA | (J.-H. Song et al., 2014) |
| Norpluviine | 84 | Lycorine-type | *L. albiflora* | Bulbs | NA | (Y. Guo et al., 2014) |
| Pluviine | 85 | Lycorine-type | *L. aurea*  *L. guangxiensis*  *L. haywardii*  *L. longituba*  *L. longituba*  *L. radiata var. pumila*  *L. sprengeri*  *L. squamigera* | Bulbs | Neuroprotective effects | (Y. Guo et al., 2014)  (An Jin et al., 2014)  (Yongqiang Tian et al., 2015b)  (X. Li et al., 2013) |
| Hippadine | 86 | Lycorine-type | *L. guangxiensis*  *L. longituba*  *L. radiata*  *L. sprengeri* | Bulbs | AChE inhibitory activity；Neuroprotective effects | (Yongqiang Tian et al., 2015b)  (Zhu et al., 2015)  (X. Li et al., 2013)  (W. M. Wu et al., 2014) |
| Lycosprenine | 87 | Lycorine-type | *L. sprengeri* | Bulbs | Neuroprotective effects | (W. M. Wu et al., 2014) |
| Sternbergine | 88 | Lycorine-type | *L. traubii* | Bulbs,  The aerial part | NA | (Toriizuka et al., 2008) |
| Tortuosine | 89 | Lycorine-type | *L. sprengeri* | Bulbs | Neuroprotective effects | (W. M. Wu et al., 2014) |
| 1-Hydroxyungeremine | 90 | Lycorine-type | *L. radiata* | Bulbs | Antitumor activity | (Z. M. Liu et al., 2015) |
| Ungiminorine | 91 | Lycorine-type | *L. incarnata* | Flowers | NA | (Kihara et al., 1994)  (Toriizuka et al., 2008) |
| Ungiminorine-N-oxide | 92 | Lycorine-type | *L. incarnata* | flowers | NA | (Kihara et al., 1994) |
| Montanine | 93 | Montanine-type | *L. albiflora*  *L. aurea*  *L. chinensis*  *L. longituba*  *L. squamigera* | Bulbs | NA | (Y. Guo et al., 2014)  (Kitajima et al., 2009) |
| Pancratinine C/Squamigine | 94 | Montanine-type | *L. longituba*  *L. radiata*  *L. squamigera* | Bulbs | NA | (Y. Guo et al., 2014)  (Kitajima et al., 2009)  (X. Li et al., 2013) |
| (−)-3-O-Menthylpancracine | 95 | Montanine-type | *L. radiata* | Bulbs | NA | (X. Li et al., 2013) |
| Pancracine | 96 | Montanine-type | *L. radiata* | Bulbs | Antiviral activity | (X. Li et al., 2013) |
| Montabuphine | 97 | Montanine-type | *L. sprengeri* | Bulbs | NA | (W. M. Wu et al., 2014) |
| Lycolongirine C | 98 | Montanine-type | *L. longituba* | Bulbs | AChE inhibitory activity | (Zhu et al., 2015) |
| 7-Deoxynarciclasine/Lycoricidine | 99 | Narciclasine-type | *L. albiflora*  *L. radiata*  *L. sanguinea*  *L. squamigera*  *L. traubii* | Bulbs | Antitumor activity | (Maki Jitsuno et al., 2011)  (Feng et al., 2011)  (Yun et al., 2016)  (Kitajima et al., 2009)  (Toriizuka et al., 2008) |
| Narciclasine/Lycoricidinol | 100 | Narciclasine-type | *L. albiflora*  *L. radiata*  *L. sanguinea*  *L. squamigera*  *L. traubii* | The whole aerial parts | Antitumor activity；Anti-inflammatory activity | (Maki Jitsuno et al., 2011)  (Yun et al., 2016)  (Kitajima et al., 2009)  (Toriizuka et al., 2008)  (X. Li et al., 2013) |
| 5,6-Dihydrobicolorine | 101 | Narciclasine-type | *L. radiata*  ***L. sprengeri*** | Bulbs | NA | (Feng et al., 2011)  (W. M. Wu et al., 2014) |
| Bicolorine | 102 | Narciclasine-type | *L. sanguinea* | Bulbs | NA | (L. Wang et al., 2010) |
| N-Methylcrinasiadine | 103 | Narciclasine-type | *L. longituba* | Bulbs | AChE inhibitory activity | (Zhu et al., 2015) |
| N-Isopentylcrinasiadine | 104 | Narciclasine-type | *L. sprengeri* | Bulbs | NA | (W. M. Wu et al., 2014) |
| Crinasiadine | 105 | Narciclasine-type | *L. sprengeri* | Bulbs | NA | (W. M. Wu et al., 2014) |
| 5,6-Dihydro-5-methyl-2-hydroxyphenanthridine | 106 | Narciclasine-type | *L. aurea*  *L. radiata* | Bulbs | Antitumor activity；Antimalarial activity | (J.-H. Song et al., 2014)  (Bin Hao et al., 2013) |
| Trisphaeridine | 107 | Narciclasine-type | *L. longituba*  *L. radiata*  *L. sprengeri* | Bulbs | AChE inhibitory activity | (Y. Guo et al., 2014)  (L. Wang et al., 2010) |
| 3-O-Ethyltazettinol | 108 | Tazettine-type | *L. aurea* | Bulbs | NA | A new alkaloid from Lycoris aurea |
| Deoxydihydrotazettine | 109 | Tazettine-type | *L. radiata* | Bulbs | aphicial activit | (Yan et al., 2018) |
| Deoxypretazettine | 110 | Tazettine-type | *L. longituba*  *L. radiata* | Bulbs | AChE inhibitory activity | (Yan et al., 2018)  (Y. Guo et al., 2014) |
| Tazettine | 111 | Tazettine-type | *L. albiflora*  *L. aurea*  *L. chinensis*  *L. haywardii*  *L. longituba*  *L. radiata var. pumila*  *L. sprengeri*  *L. squamigera* | Bulbs,  Flowers | In vitro antimalarial activity | (Y. Guo et al., 2014)  (Kitajima et al., 2009) |
| 6-O-Methylpretazettine | 112 | Tazettine-type | *L. squamigera* | Bulbs | NA | (Kitajima et al., 2009) |
| 3-Epimacronine | 113 | Tazettine-type | *L. radiata* | Bulbs | Aphicial activity | (Yan et al., 2018) |
| 3-Hydroxylatifaliumin C * | 114 | Tazettine-type | *L. guangxiensis*  *L. radiata* | Bulbs | NA | (Yongqiang Tian et al., 2015b) |
| Dihydrolatifaliumin C * | 115 | Tazettine-type | *L. aurea*  *L. guangxiensis*  *L. radiata* | Bulbs | NA | (Yongqiang Tian et al., 2015b) |
| Latifaliumin C * | 116 | Tazettine-type | *L. aurea* | Bulbs | NA | (Yongqiang Tian et al., 2015b) |
| Norharmane | 117 | Other structural types | *L. longituba* | Bulbs | NA | (Zhu et al., 2015) |
| Harmane | 118 | Other structural types | *L. longituba* | Bulbs | NA | (Zhu et al., 2015) |
| Lycolongirine A | 119 | homolycorine-type alkaloid | *L. longituba* | Bulbs | NA | (Zhu et al., 2015) |
| Perlolyrine | 120 | Other structural types | *L. longituba* | Bulbs | NA | (Zhu et al., 2015) |
| Colchicine | 121 | Other structural types | *L. radiata* | Bulbs | NA | (Yan et al., 2018) |
| N-Methoxycarbonyl-2-demethyl-isocorydione | 122 | Other structural types | *L. radiata* | Bulbs | Anti-inflammatory activity | (Z. M. Liu et al., 2015) |
| 2-demethyl-isocorydione | 123 | Other structural types | *L. aurea* | Bulbs | Anti-inflammatory activity | (J.-H. Song et al., 2014) |
| Isocorydione | 124 | Other structural types | *L. aurea* | Bulbs | Anti-inflammatory activity | (J.-H. Song et al., 2014) |
| 8-demethyl-dehydrocrebanine | 125 | Other structural types | *L. aurea* | Bulbs | Anti-inflammatory activity | (J.-H. Song et al., 2014) |
| (+)-N-methoxylcarbonyl-nandigerine | 126 | Other structural types | *L. aurea* | Bulbs | Antitumor activity | (Peng Cao et al., 2013) |
| (+)-N-methoxycarbonyl-lindcarpine | 127 | Other structural types | *L. aurea* | Bulbs | Antitumor activity | (Peng Cao et al., 2013) |
| 10-O-Methylhernovine-N-oxide | 128 | Other structural types | *L. aurea* | Bulbs | Antitumor activity | (Peng Cao et al., 2013) |
| Hemanthamine | 129 | Haemanthamine-type | *L.radiata* | Bulbs | Antiviral activity | (J. He et al., 2013) |
| hemanthidine | 130 | Haemanthamine-type | *L. radiata* | Bulbs | Antiviral activity | (Yeo et al., 2021) |
| N-methyl-2,3,4-trimethoxylycoricidine | 131 | Lycorine-type | *L. radiata* | Bulbs | Antiviral activity | (D. Q. Yang et al., 2018) |
| *N*-methyl-2-methoxy-3,4-acetonidelycoricidine | 132 | Lycorine-type | *L. radiata* | Bulbs | Antiviral activity | (D. Q. Yang et al., 2018) |
| (+)-8-hydroxy-homolycorine-α-N-oxide | 133 | Homolycorine-type | *L. aurea* | Bulbs | Antitumor activity | (J. H. Song et al., 2014) |
| Narciclasine | 134 | Narciclasine-type | *L. radiata* | Bulbs | Antifungal activity | (Qiao et al., 2023) |
| 11-hydroxy vittatine | 135 | Crinine-type | *L.radiata* | Bulbs | Antiviral activity | (J. He et al., 2013) |
| Lycoricidinoal | 136 | Lycorine-type | *L. radiata* | Bulbs | NA | (Yeo et al., 2021) |
| 1-hydroxy-ungeremine | 138 | Lycorine-type | *L. radiata* | Bulbs |  | (Z. M. Liu et al., 2015) |
| N-methoxycarbonyl-2-demethyl-isocorydione | 137 | Galanthamine-type alkaloids | *L. radiata* | Bulbs | Anti-inflammatory activity | (Z. M. Liu et al., 2015) |
| 7-deoxy-trans-dihydronarciclasine | 138 | Lycorine-type Alkaloid | *L. chejuensis* | Bulbs | NA | (Lucie Cahlíková et al., 2020) |
| Yemenine A | 139 | Galanthamine-type Alkaloid | *L. radiata* | Bulbs |  | (Hu et al., 2018) |

* This table summarizes the alkaloid-rich species within the *Lycoris* genus, along with the names of identified alkaloids, their tissue-specific distribution, and documented pharmacological activities. Current research indicates that the majority of these alkaloids are predominantly localized in the bulbs, whereas their presence and profiles in leaves, root hairs, and flowers remain less explored. "NA" denotes that the pharmacological activity for the corresponding alkaloid has not been reported in the extant literature.
